# Carbenoxolone induced depression of rhythmogenesis in the pre-Bötzinger Complex

**DOI:** 10.1186/1471-2202-9-46

**Published:** 2008-05-23

**Authors:** Frank P Elsen, Edward J Shields, Matthew T Roe, Richard J VanDam, Jonathan D Kelty

**Affiliations:** 1 Department of Neurology, Medical College of Wisconsin, Milwaukee, WI 53226, USA; 2 Department of Biology, Central Michigan University, Mt. Pleasant, MI 48859, USA

## Abstract

**Background:**

Carbenoxolone (CBX), a gap junction uncoupler, alters the functioning of the pre-Bötzinger Complex (preBötC), a central pattern generating neuronal network important for the production of respiratory rhythm in mammals. Even when isolated in a 1/2 mm-thick slice of medulla oblongata from neonatal mouse the preBötC continues producing periodic bursts of action potentials, termed population bursts that are thought to be important in generating various patterns of inspiration, *in vivo*. Whether gap junction communication contributes to preBötC rhythmogenesis remains unresolved, largely because existing gap junction uncouplers exert numerous non-specific effects (e.g., inhibition of active transport, alteration of membrane conductances). Here, we determined whether CBX alters preBötC rhythmogenesis by altering membrane properties including input resistance (R_in_), voltage-gated Na^+ ^current (I_Na_), and/or voltage-gated K^+ ^current (I_K_), rather than by blocking gap junction communication. To do so we used a medullary slice preparation, network-level recordings, whole-cell voltage clamp, and glycyrrhizic acid (GZA; a substance used as a control for CBX, since it is similar in structure and does not block gap junctions).

**Results:**

Whereas neither of the control treatments [artificial cerebrospinal fluid (aCSF) or GZA (50 μM)] noticeably affected preBötC rhythmogenesis, CBX (50 μM) decreased the frequency, area and amplitude of population bursts, eventually terminating population burst production after 45–60 min. Both CBX and GZA decreased neuronal R_in _and induced an outward holding current. Although neither agent altered the steady state component of I_K _evoked by depolarizing voltage steps, CBX, but not GZA, increased peak I_Na_.

**Conclusion:**

The data presented herein are consistent with the notion that gap junction communication is important for preBötC rhythmogenesis. By comparing the effects of CBX and GZA on membrane properties our data a) demonstrate that depression of preBötC rhythmogenesis by CBX results from actions on another variable or other variables; and b) show that this comparative approach can be used to evaluate the potential contribution of other non-specific actions (e.g., Ca^++ ^conductances or active transport) of CBX, or other uncouplers, in their alteration of preBötC rhythmogenesis, or the functioning of other networks.

## Background

Located within the ventrolateral medulla the preBötC is a central pattern generating neuronal network that rhythmically produces bursts of action potentials that are important for respiratory rhythmogenesis [1-3]. Regarding inter-cellular communication, most research on respiratory rhythmogenesis has focused on chemical synaptic transmission and neuromodulation [4-11]. Recent research has begun examining the potential contribution of electrical and cytoplasmic coupling via gap junctions in the functioning of central respiratory networks [12-19]. Mammalian gap junctions, like ion channels, are multi-unit structures of integral membrane proteins [20,21]. The best studied of these proteins are connexins (Cx), although pannexins are also expressed in mammals [22,23]. A connexin-based gap junction channel is composed of two hemi-channels, or connexons, that together span the membranes of adjacent cells. Each connexon is composed of six Cx subunit proteins, each with four transmembrane domains, three intracellular regions (the amino terminus, carboxy-terminus, and a cytoplasmic loop), and two extracellular loops [20,24-26].

Multiple lines of evidence suggest that gap junction connectivity is important within the medullary region containing the preBötC. Immunohistochemical and immunoblot studies indicate that neurons within the preBötC, as well as within other regions at the same rostro-caudal level of the medulla oblongata (e.g., XII nucleus, Inferior Olivary Complex), express connexins of the 26, 32, and 36 kDa families, termed Cx26, Cx32 and Cx36, respectively [14,27]. A study using reporter genes and *in situ *hybridization supports the finding that Cx36 is expressed by neurons in the region of the preBötC [28]. Gap junction uncouplers such as CBX, 18α-glycerrhetinic acid (18α-GA), 18β-glycerrhetinic acid (18β-GA), heptanol, or octanol change the frequency and pattern of respiratory network burst generation [16,19,29,30], even to the point of terminating preBötC rhythmogenesis after an hour of exposure to CBX [29]. Dual intracellular recordings demonstrate that inspiration related neurons in the preBötC and nucleus ambiguous (NA) are electrically coupled [29,31].

The aforementioned evidence notwithstanding, the question of whether gap junctions have a functional role in preBötC rhythmogenesis remains unresolved. Expression of Cx mRNA or protein does not demonstrate the presence of functional gap junctions. Even when electrical coupling has been demonstrated between preBötC neurons, the coupling ratio between neurons was found to be low [29]. Data suggesting that pharmacological manipulation of gap junctions affects the functioning of rhythmogenic networks must be interpreted cautiously; gap junction uncouplers are notorious for the broad range actions other than blockade of gap junction functionality [32,33]. By example, CBX, perhaps the most widely used uncoupler, attenuates transmembrane conductances including Ca^++ ^conductances in photoreceptors [32], decreases the excitability of cultured neurons [33], inhibits 11β-hydroxysteroid dehydrogenase in arterial endothelial cells [34], may trigger release of NO from arterial endothelium [35] and induces oxidative stress in liver mitochondria [36,37]. In the preBötC, CBX decreases neuronal input resistance and action potential production in response to depolarizing current pulses [29].

As CBX exerts a variety of non-specific effects on neurons, others have proposed that CBX may affect preBötC rhythmogenesis through such actions, rather than by blocking gap junction communication [29]. Because GZA is similar in structure to CBX and is known to evoke a number of the same cellular responses, but without blocking gap junction connectivity, others have used it as a control for CBX when examining the effects of CBX on pre-BötC functionality at the network level [16,19]. However, in the preBötC at least, GZA has not been used to control for the actions of CBX on discreet membrane properties. Here we characterize the effects of CBX on membrane properties (input resistance (R_in_), holding current (I_hold_) at -60 mV, voltage-gated sodium current (I_Na_), and steady-state voltage-gated potassium current (I_Kd_)) of preBötC neurons. Our data are consistent with the hypothesis that gap junctions are important to the rhythmic production of inspiration-related neuronal activity by the preBötC. That is, by comparing the population-level and single-cells effects of CBX with those of GZA, we demonstrate that while CBX alters and even terminates preBötC rhythmogenesis it does so without significantly affecting a number of critical neuronal variables.

## Results

### Effects of CBX and GZA on population activity

**Figure 1:**
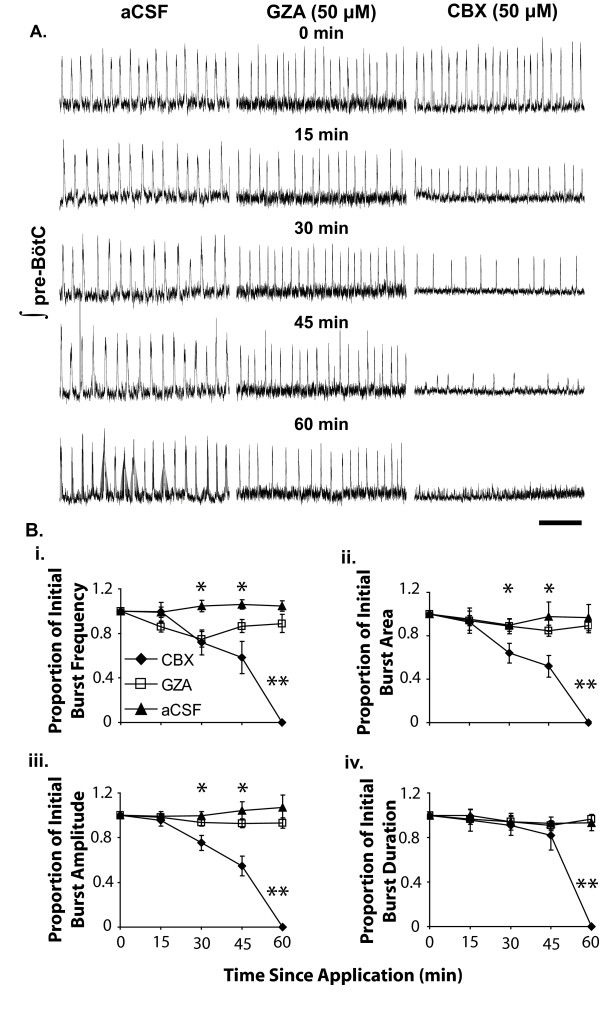
**Effects of CBX and GZA on population-level bursting by the preBötC**. **A**. Sample traces of extracellular activity recorded and integrated using a 50 ms time constant in the presence of GZA or CBX. Time bar represents 20 seconds. **B**. Whereas CBX decreased mean burst frequency within 20 min of application (i.), slices bathed in aCSF containing 50 μM GZA continued bursting at or near baseline frequency throughout recording (Two-way RM ANOVA with treatment by time as the source of variation d.f. = 4, F = 24.025, P < 0.001). B.ii. CBX decreased the area of integrated population bursts (Two-way RM ANOVA with treatment by time as the source of variation d.f. = 4, F = 25.783, P < 0.001). B.iii., Decreased burst area with CBX treatment appears to have been due to CBX decreasing burst amplitude beginning at 30 min of treatment (Two-way RM ANOVA with treatment by time as the source of variation d.f. = 4, F = 59.14, P < 0.001), as burst duration was seemingly unaffected by CBX up to the point at which bursting ceased (B.iv). *CBX value different from aCSF control at P < 0.05. **In these figures, the 60 min data point is presented to underscore that CBX terminated preBötC bursting within 1 h of treatment.

Earlier studies show that the specific effects of gap junction uncouplers on preBötC-related outputs (e.g., XII nerve rootlet activity) vary between species, age of organism, and between types of preparation [16,17,19,30]. Thus, the initial part of this study determined whether medullary tissue from neonatal mice responded to CBX, a commonly-used gap junction uncoupler, in the same manner as it has been reported in previous studies [16,17,19,30]. Within 30 minutes of exposure, CBX (50 μM) decreased population burst frequency by 25.5% from 0.31 ± 0.05 to 0.23 ± 0.06 Hz (n = 10, p = 0.01, Tukey Test; Fig. 1). After 45 minutes of CBX exposure burst frequency decreased by 47% to 0.16 ± 0.05 Hz and by 1 hour CBX had completely suppressed bursting in all slices used in this study (n = 10).

Two types of controls were used in this study: Slices treated with GZA (50 μM) and slices kept in aCSF alone (no drug applications) throughout the experiments. Although GZA has been reported to affect some of the same aspects of cellular function as CBX, it produced no detectable effect on burst frequency, except at 30 minutes, when it appeared to cause a transient ~33% dip in population burst frequency from 0.31 ± 0.06 to 0.21 ± 0.03 Hz (Fig 1B.i). Slices kept in aCSF with no drug addition for the same period as those treated with CBX or GZA showed no significant change in population burst frequency (0.28 ± 0.06 Hz at the beginning of observation compared to 0.30 ± 0.07 Hz one hour later).

Although inhibition of neuronal/neuronal network function caused by short term exposure to CBX is reversible [38], CBX may be cytotoxic at concentrations ≥ 100 μM, or when applied for long periods (e.g, ≥ 24 h) [34]. Accordingly, washout experiments were used to determine whether suppression of preBötC rhythmogenesis by CBX could be reversed. Within 45–60 min after the beginning of the washout period, 4 of 5 slices resumed bursting. Prior to CBX treatment the preBötC generated bursts at 0.20 ± 0.03 Hz. During the period between 55 and 60 min following the initiation of washout slices generated bursts at 0.10 ± 0.02 Hz (n = 4 slices, P = 0.12, Paired t-test).

In contrast to both control conditions (no drugs or GZA treatment), CBX decreased the area of population bursts (Fig. 1B.ii). After 30 min of treatment, CBX decreased population burst area by 40% from 1.3e^-3 ^to 7.9e^-4 ^μV·s^2 ^(Tukey test: n = 10, P < 0.001). Burst area remained relatively unchanged for slices kept in aCSF or in GZA. As bursting ceased by 1 hr of treatment with CBX, there was no burst area to measure at this time point. By contrast, even after 1 hr of recording, slices in aCSF continued to generate bursts with 96.7 ± 12.1% the area observed during initial (baseline) recording. Similarly, after 1 hr of recording, slices in GZA continued to generate bursts with 88.6 ± 15.4% the area observed during baseline recording.

CBX decreased burst area by affecting burst amplitude, rather than burst duration. After 30 minutes of CBX treatment, burst amplitude had decreased by 25% from 3.0e^-3 ^μV·s to 2.3e^-3 ^μV·s (Tukey test: n = 10, P < 0.001), and after 45 minutes by 46% to 1.6e^-3 ^μV·s (Tukey test: n = 10, P < 0.001 Fig. 1B.iii). In contrast, burst duration remained relatively constant throughout the treatment with CBX (start: 559.3 ± 41.5 ms, 45 min: 475.0 ± 29.1 ms) (Tukey test: n = 10, P = 0.139; Fig 1B.iv). Whereas 1 hr of CBX treatment stopped burst generation, the burst amplitude and duration of slices in aCSF and GZA remained indistinguishable form baseline levels throughout recording. After 1 hr, slices in aCSF continued generating bursts with a mean duration and amplitude of 530.8 ± 39.0 ms and 2.6e^-3 ^μV·s^2^, respectively (vs. 587.7 ± 55.1 ms and 2.4e^-3 ^μV·s^2 ^during baseline recording).

### Effects of CBX and GZA on Whole-Cell Properties

Prior studies [29] within the preBötC examined the actions of CBX on single neuron/membrane properties, without comparing these actions to those of a control agent (other than aCSF). In this study we did so using whole cell voltage clamp. Whole-cell voltage clamp measurement often remained stable for up to an hour with cells exposed to normal aCSF or GZA. However, in the presence of CBX few whole-cell recordings lasted for more than 20 min.

It has been reported in an earlier study [29] that in preBötC neurons, CBX alters passive membrane properties and thereby decreases neuronal excitability. We found that R_in _of both CBX and GZA treated neurons progressively decreased over time (Table 1). By contrast, R_in _remained similar to its initial value for neurons in slices exposed to aCSF (without CBX or GZA) for the same duration as those exposed to CBX or GZA. Over a period of 20 min the I_hold _of neurons monitored in aCSF changed little (Table 1). In contrast, GZA and CBX both increased the magnitude of the negative holding current (Table 1).

**Table 1:** Effects of aCSF, GZA and CBX treatment on resting membrane properties of preBötC neurons.

**Condition**	**n**	**Input Resistance**^1^**(R**_in_**; MΩ)**	**Holding Current^2^(_Ihold_; pA)**
aCSF			
0 min	10	339.1 ± 66.4	-60.8 ± 14.9
5 min	10	362.8 ± 126.1	-73.7 ± 14.9
10 min	10	327.2 ± 63.4	-71.2 ± 14.9
15 min	10	358.1 ± 122.1	-73.7 ± 13.5
20 min	10	329.2 ± 82.4	-82.7 ± 13.6
GZA (50 μM)			
0 min	11	308.2 ± 66.2	-67.3 ± 8.4
5 min	11	232.4 ± 91.9	-110.4 ± 29.8
10 min	11	212.0 ± 41.2	-125.8 ± 25.9
15 min	11	182.9 ± 79.3	-160.8 ± 31.9
20 min	11	160.0 ± 29.5	-200.0 ± 38.7
CBX (50 μM)			
0 min	8	387.6 ± 48.5	-53.9 ± 7.9
5 min	8	238.7 ± 41.5	-73.8 ± 7.3
10 min	8	260.3 ± 78.1	-116.3 ± 29.8
15 min	7	237.7 ± 56.0	-99.4 ± 14.4
20 min	7	197.2 ± 40.4	-168.9 ± 48.7

All values shown were obtained from neurons in the presence of CdCl_2 _and comparison was performed between values for respective time intervals.

^1^Input resistance decreased for both CBX- and GZA-treated neurons over time (Friedman's Repeated Measures ANOVA on ranks for CBX: df = 4, SS = 393977.8, MS = 98494.5, F = 2.798, P = 0.040; Friedman's Repeated Measures ANOVA on ranks for GZA: df = 4, SS = 137567.026, MS = 34391.757, F = 3.457, P = 0.023).

^2^Compared using Kruskal-Wallace ANOVA on Ranks (H = 44.93, d.f. = 14, P < 0.001).

**Figure 2:**
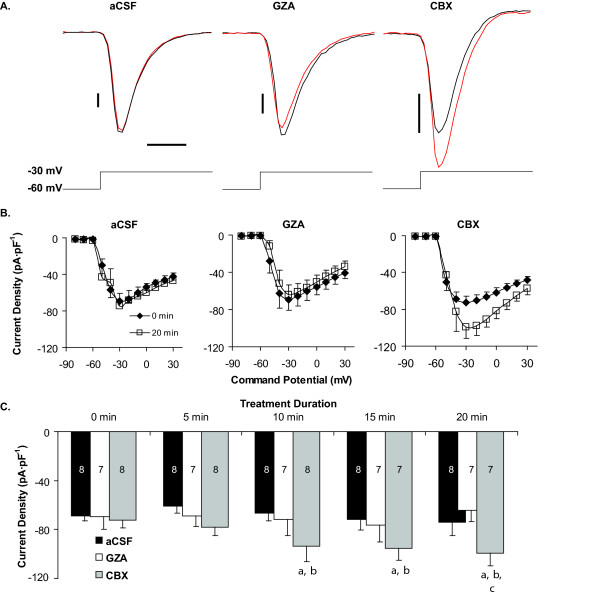
**Glycyrrhizic acid and Carbenoxolone differentially affect I_Na_**. A. Representative Traces from whole-cell voltage clamp experiments demonstrating the lack of change in peak I_Na _over time in aCSF or GZA (50 μM) *vs*. increased peak I_Na _in CBX (50 μM). Black trace represents control conditions and red trace represents drug effect. Vertical scale bars = 1 nA; time bar represents 1 ms. B. Current-Density *vs*. voltage relationships under baseline (t = 0 min) and after 20 min in the indicated treatment. Whereas GZA (50 μM) produced no consistent change in I_Na _CBX (50 μM) increased the density of this current. C. Current density evoked during voltage steps from -60 to -30 mV. a: different from baseline (t = 0 min) at P < 0.05; b: different from aCSF at P < 0.05; c: different from GZA at P < 0.05. The numbers within each column represent sample size.

Little is known about the effects of CBX on voltage-gated conductances, therefore we tested whether CBX would alter transient voltage-gated sodium currents (I_Na_) and steady state potassium currents (I_Kd_) using standard voltage-step protocols. Under control conditions I_Na _did not significantly change throughout the duration of the experiments (*ca*. 20 min). In contrast, I_Na _density of neurons treated with CBX progressively increased over time. At the command voltage evoking the peak I_Na _(-30 mV), CBX significantly increased I_Na _density after 10 minutes from -73.5 ± 8.8 pA·pF^-1 ^to -93.8 ± 18.7 pA·pF^-1 ^(n = 7, P < 0.05) and after 20 minutes to -111.6 ± 19.12 (P = 0.023). By contrast, GZA treatment caused no significant change in I_Na _or I_Na _density (Fig. 2).

**Figure 3:**
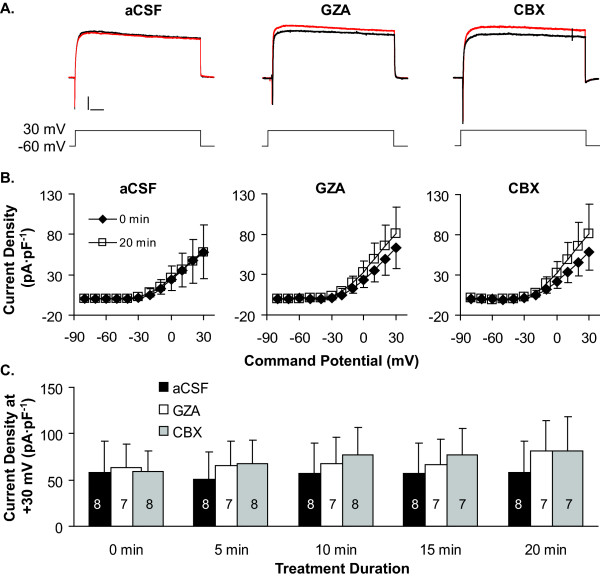
**Steady-state I_K _(I_Kd_) remains near baseline values during treatment with Glycyrrhizic acid or Carbenoxolone**. A. Representative whole cell voltage clamp traces demonstrating the lack of significant change in I_K _over time (20 min) in aCSF or GZA (50 μM). Horizontal scale bar = 20 ms; vertical scale bar = 1 nA. Black trace represents control conditions and red trace represents drug effect. B. Current Density *vs*. voltage relationships during the baseline (t = 0 min) sample and after 20 min in the indicated treatment. C. Current density evoked during voltage steps from -60 to 30 mV remained similar to baseline value during treatment with CBX or GZA. The numbers within each column represent sample size.

Voltage-gated potassium currents were not detectably affected by CBX or GZA. For example initially, a voltage step from -60 mV (V_hold_) to a command potential of 30 mV evoked I_Kd _at a current density of 58.7 ± 29.31 pA·pF^-1 ^After 20 minutes of CBX application, from the same voltage step evoked I_Kd _at a similar density (78.1 ± 45.1 pA·pF^-1^; Fig. 3, n = 7, P > .05). The density of I_Kd _of GZA-treated neurons after 20 minutes of treatment (67.9 ± 39 pA·pF^-1^) was similar to that observed initially (67.9 ± 39 pA·pF^-1^; Fig. 3, n = 7, P > 0.05).

## Discussion

The fact that gap junction uncouplers evoke numerous cellular responses in addition to blocking gap junction communication has impeded efforts to determine the extent to which gap junction communication may contribute to preBötC rhythmogenesis. Nonetheless, it remains unclear as to when or whether gap junction blockers that don't otherwise interfere with neuronal functionality will be developed and/or become commercially available. The data presented herein extend prior work in characterizing previously undescribed effects of CBX on transmembrane currents of preBötC neurons. By comparing the effects of CBX on individual preBötC neurons with those of the control agent, GZA our data provide direction and the initial steps in clarifying the ambiguity concerning how CBX affects network level function.

In this study substantial effort was put forth to determine the effects of CBX on rhythmogenic activity of the preBötC and to qualitatively compare these data with those of prior studies [16,18,19,29,30]. Doing so was important at this stage in our research program as the importance of gap junctions, or at least the effects of uncouplers, appears to vary from one description to another. For instance, whereas CBX, 18α-GA and 18β-GA decrease the frequency of phrenic nerve rootlet inspiratory activity observed using *en-bloc *brainstem preparations and medullary slice preparations from neonatal Swiss-Webster mice [30] these agents increase phrenic burst frequency observed using *in vitro *arterially perfused rat preparations [16]. Rekling and colleagues [29], using the medullary slice preparation from neonatal mice found that simply extending the period of CBX exposure to 45–60 min totally suppressed breathing-related output. By contrast, gap junction uncouplers administered arterially to *in situ *preparations from juvenile rat produce no consistent effect on eupneic or gasp inspiration patterns [18]. Yet others have demonstrated increases in eupneic phrenic burst frequency and modulation of the eupneic phrenic burst pattern with *in situ *preparations from rat [16,19]. Our extracellular data are similar to those of others using the medullary slice preparation from juvenile mice [29,30].

The medullary slice preparation used for this study is vastly more reduced than most of the other preparations used to study respiratory rhythmogenesis (e.g., the brainstem spinal cord preparation or the working heart brainstem preparation) [39,40]. Nonetheless, the ~500 μm-thick slice likely contains portions of adjacent regions, including the Bötzinger complex and rostral Ventral Respiratory Group [41], that could potentially modulate preBötC rhythmogenesis [42]. Thus, it is conceivable that application of uncouplers to the slice preparation could affect preBötC rhythmogenesis not by acting directly on the preBötC, but indirectly by actions on neurons in nearby regions that in turn modulate the activity of the preBötC.

Knowing that gap junction uncouplers tend to exert a variety of non-specific effects, ideally their use would be coupled to the use of an appropriate control agent; that is, a substance that mimics as many of the non-specific actions of the uncoupler as possible, without blocking gap junction communication. Both CBX and GZA elicit at least some of the same cellular responses. For instance, CBX and GZA have been reported to attenuate Ca^++ ^currents, albeit in separate tissues [32,43]. However, only a small proportion of studies examining the potential role of gap junction communication in preBötC rhythmogenesis employ control agents, such as GZA as a control for CBX, or heptanol, as a control for octanol [16]. None use GZA to control for the actions of CBX examined at the level of individual preBötC neurons.

Consistent with the decreased neuronal excitability attributed to CBX in prior work [29] CBX in the present study decreased R_in_. However, whereas such actions led prior workers to deem the network level effects of CBX inconclusive [29], the use of GZA as a control in this study suggests that the effects of CBX on resting membrane properties may not be sufficient to affect the rhythmic output of the preBötC observed at the population level. This result should not be interpreted as a suggestion that CBX fails to affect other variables related to neuronal excitability, only that since both CBX and GZA decrease R_in _and evoke an outward holding current in preBötC neurons voltage clamped at -60 mV CBX must affect network level output by affecting another mechanism or mechanisms. Interestingly, whereas others found that CBX causes neuronal hyperpolarization we found that both CBX and GZA induced an inward holding current relative to neurons kept in aCSF. As access resistance was monitored throughout experimentation and only samples for which this variable was both less than 40 MΩ and less than 0.1 times the R_in _were used, it seems unlikely that such a striking increase would reflect deterioration of the seal between the membrane and the patch pipette. Both CBX and GZA affect active transport and in so doing could lead to longer term changes in ionic equilibria [37]. Regardless, as both CBX and GXA induced similar changes in I_hold_, it is unlikely that the effects of CBX on I_hold _contribute to its overall effects on preBötC rhythmogenesis.

Another possibility is that CBX decreases preBötC rhythmogenesis not by blocking gap junction communication, but rather by affecting voltage-gated conductances. This study examined potential effects of CBX and GZA on I_Na _and I_Kd_. Neither CBX nor GZA affected I_Kd_, showing that alteration of (non-inactivating) voltage-gated potassium conductances does not contribute to the alteration of preBötC rhythmogenesis by CBX. By contrast, whereas CBX steadily increased the density of I_Na_, GZA produced no detectable change in this variable. However, it is unclear whether such a change would lead to the network-level effects associated with CBX. In retinal photoreceptors CBX decreases Ca^++ ^currents [32]. In the preBötC such changes could impede burst production by one subset of pacemaker neurons, the calcium-dependent pacemakers [44,45]. Attenuation of Ca^++ ^currents would also depress (chemical) synaptic transmission, thereby depressing network-level output. It must be noted that rather than identifying distinctly respiration-related neurons, this study focused on all neurons within the preBötC.

Accordingly, upcoming studies will explore potential effects of CBX and GZA on a variety of voltage gated Ca^++ ^currents in inspiration-related preBötC neurons.

## Conclusion

The data presented herein represent an important step in resolving the issue of whether or not gap junction communication is important to preBötC rhythmogenesis. Rhythmic output generated by the population of preBötC neurons was depressed by CBX but not GZA. In contrast, both CBX and GZA affected resting membrane properties in the same way, and neither affected I_Kd _suggesting that the network level effects of CBX are caused by it affecting one or more other cellular actions. In that our data demonstrate CBX affects a number of membrane properties in the same manner as GZA, which does not affect network-level output, they are consistent with the notion that CBX inhibits rhythmogenesis by interfering with gap junction coupling between preBötC neurons. However, the non-specific actions of CBX examined herein represent only a subset of those attributed to CBX. To completely evaluate the notion that CBX affects preBötC rhythmogenesis by uncoupling gap junction channels, future research will need to evaluate the actions of CBX on numerous neuronal variables (e.g., Ca^++ ^channels and fast chemical synaptic transmission). However, characterizing the actions of CBX on such variables will not, on its own, demonstrate conclusively that any one of those properties, or any combination of those properties, underlies the actions of CBX on network-level output. As shown here, applying the most fundamental of all scientific principles, the use of an appropriate control, provides the opportunity to potentially falsify the notion that CBX-induced changes in a given neuronal property cause its network level effects. Using this approach can thus strengthen one's confidence that CBX-induced changes in a variable cause, or are unrelated to its network level effects.

## Methods

### Medullary slice preparation

All procedures were carried out according to guidelines established by the National Institutes of Health and National Research Council (U.S.A.) and were approved by the Institutional Animal Care and Use Committee at Central Michigan University (protocol # 23-04). Functionally-intact slices of medulla oblongata containing the preBötC were obtained from male and female Swiss-Webster mice (postnatal days 3–7) decapitated at the C3/C4 vertebrae level according to the methods outlined by Ramirez and colleagues [46]. In brief: The brainstem was isolated in ice-cold artificial cerebrospinal fluid (aCSF; containing in mM: 118 NaCl, 3 KCl, 1.5 CaCl_2_, 1 MgCl_2_, 25 NaHCO_3_, 1 NaH_2_PO_4_, and 30 D-Glucose) infused with carbogen gas (95% O_2 _and 5% CO_2_). The isolated brainstem was secured to an agar block and serial slices (*ca*. 250 μm-thick) were removed from the rostral end using a vibrating microtome (VT 1000, Vibratome, Inc., St. Louis, MO). Once the level of the preBötC was revealed, as recognized by the appearance of landmarks including the IO (inferior olive), the NA (nucleus ambiguous), and the XII nucleus (hypoglossus motor nucleus), a 500 μm-thick slice was cut, transferred into a recording chamber, perfused with carbogen-infused aCSF (30°C; pH 7.4) and allowed to equilibrate for 30 min prior to any subsequent treatment. Extracellular potassium concentration was raised to 8 mM for the duration of the experiments.

### Extracellular recordings

Neuronal activity (action potential production) generated by the mixed population of preBötC neurons on one side of the slice preparation was monitored and recorded using large bore glass pipettes (tip diameter *ca*.100 μm) filled with aCSF and connected via a silver chloride-coated wire to a homemade AC-coupled preamplifier (100×). The pre-amplified signal was further amplified 100 times (Amplifier model P15, Grass Technologies, West Warwick, RI, U.S.A.) and integrated with a homemade hardware integrator (50 ms time constant). The amplified trace and its integrated representation were recorded on a hard disk of a personal computer. Recording was continuous throughout consecutive periods of baseline observation, 1.25 hours of treatment with CBX, GZA or aCSF (as a time-matched control with no agents added) and up to 1.25 hours of washout. At 15 minute intervals within each treatment, samples of population bursts (2 min-long) were analyzed for burst amplitude, duration, area and frequency (Igor Pro v. 4.0; Wavemetrics, Inc.).

### Whole cell voltage clamp technique

As with population-level recordings, individual preBötC neurons were monitored in normal aCSF and in the presence of either CBX [50 μM] or GZA [50 μM]. Whole-Cell recordings [47] were obtained using unpolished electrodes fabricated from thick-walled borosilicate glass (Warner Instruments, # GC 150-10) and current amplitudes were assessed at the peak inward current (I_Na_)and steady state outward current (I_Kd_). The pipette solution contained (in mM): 140 K-Methane Sulfonate 1 CaCl_2_, 2 MgCl_2_, 4 Na_2_ATP, 0.3 GTP, 10 EGTA, and 10 HEPES; pH 7.2. Using high magnification infra-red Normarski differential interference contrast optics the tip of a patch electrode was gently pressed against the somatic neurolemma of a neuron within the preBötC. A gigaohm seal was established by gently applying negative pressure (*ca*. -20 mmHg). Once a cell-attached configuration was established, the membrane within the tip of the pipette was ruptured by applying repeated pulses of negative pressure. Current traces were recorded either with an EPC 8/ITC 8 amplifier/data acquisition board combination (Heka Instruments, Southboro, MA, U.S.A.) or with an Axopatch 200B/Digidata 1320 amplifier/acquisition board combination (Molecular Devices Corporation, Sunnyvale, CA, U.S.A.). Current traces were filtered at 2 kHz using the internal bessel filter of the respective amplifier and digitized at10 kHz. Data were recorded with either Patchmaster 2.1 software (Heka Intruments) or pClamp 9.2 (Molecular Devices Corporation) and stored on a personal computer hard-drive. Prior to the data recording, transient currents caused by uncompensated pipette capacitance, series resistance and membrane capacitance were minimized, and the serial resistance was 80% compensated and adjusted throughout the experiments. To determine the quality of the recording, we routinely monitored vital configuration parameters throughout baseline and experimental conditions. Cell properties including R_in_, access resistance (R_a_), I_hold _at -60 mV and cell capacitance were determined with a build-in software step protocol. Voltage-gated currents were evoked with the following voltage step protocol: From a holding potential of -60 mV we applied 150 ms long voltage steps from -80 to 30 mV in 10 mV steps. Linear leak currents were eliminated with an online P/4 leak subtraction protocol.

During voltage clamp experiments, we eliminated voltage-gated calcium currents by bath application of 200 μM CdCl_2 _to pharmacologically isolate voltage gated I_K_, prior to drug application. During the chemical bath applications we monitored the current amplitudes with a chain of single voltage steps (from -60 to 10 mV, P/4 enabled, interval 10 s) to determine the time course change of voltage-gated current amplitudes. The effect of CdCl_2 _reached a steady state level within 3 minutes after start of the bath application.

### Drug application and washout

CBX and GZA were prepared by diluting stock solutions (1:1000) in the superfusion bath reservoir, and applied at their final concentration (50 μM) via a gravity driven superfusion bath. In some extracellular experiments CBX was washed out by superfusing the slice preparation for up to 1.25 h with aCSF (containing 8 mM KCl) that had been equilibrated with carbogen during the preceding CBX treatment.

### Data analysis

Only slices that generated population bursts at 0.2 – 0.4 Hz under baseline conditions were used in this study. Nonetheless the network activity of the preBötC could vary over the course of each experiment. We performed aCSF control experiments only for the population experiments. Drug induced changes of network activity parameters (burst frequencies, areas, amplitudes and durations) were normalized relative to values observed during the baseline period that is during the period immediately prior to CBX or GZA application, or prior to the control period in aCSF without either of these agents. Statistical analyses were performed before and in 15 minute intervals after the drug application. To eliminate variability caused by cell surface area, we used current densities instead of current amplitudes to determine if CBX or GZA had an effect on voltage-gated I_Na _or I_K_. Current densities were calculated by dividing current amplitude with cell capacitance. To evaluate drug induced changes we normalized the density values after drug application to the values under control conditions after bath application of CdCl_2_.

All data were tested for normality using the Kolmogorov-Smironov test. If distributed normally, population burst parameters were analyzed using repeated-measures analysis of variance. We used the Tukey post-hoc test to evaluate differences between individual treatments. When population burst data were distributed non-parametrically, we analyzed treatment effects using Freidman's repeated measures ANOVA on ranks followed by Tukey post-hoc tests. Intracellular data were compared using one-way ANOVA followed by Tukey post-hoc tests, if distributed normally. Non-normal intracellular data were evaluated using Kruskal-Wallace ANOVA, followed by Dunn's multiple comparison's test. Differences between treatments were considered significant at p = 0.05.

## Authors' contributions

FPE conducted a substantial portion of the voltage clamp recordings and made important contributions to writing the manuscript. EJS and RJVD each performed some of the voltage clamp recordings and provided input on the design of this portion of the project. MTR collected most of the extracellular data and helped draft an early version of the manuscript that primarily dealt with this data. JDK conceived of the project, obtained funding for the project, conducted statistical analyses, and was the main author in writing the manuscript. All authors approved the final manuscript.
